# HPLC and Anti-Inflammatory Studies of the Flavonoid Rich Chloroform Extract Fractionof *Orthosiphon Stamineus* Leaves

**DOI:** 10.3390/molecules15064452

**Published:** 2010-06-21

**Authors:** Mun Fei Yam, Vuanghao Lim, Ibrahim Muhammad Salman, Omar Ziad Ameer, Lee Fung Ang, Noersal Rosidah, Muthanna Fawzy Abdulkarim, Ghassan Zuhair Abdullah, Rusliza Basir, Amirin Sadikun, Mohd Zaini Asmawi

**Affiliations:** 1 School of Pharmaceutical Sciences, Universiti Sains Malaysia, 11800 Minden, Pulau Pinang, Malaysia; E-Mails: amirin@usm.my (A.S.); amzaini@usm.my (M.Z.A.); 2 Department of Anatomy, Faculty of Medicine and Health Science, Universiti Putra Malaysia, 43000 Serdang, Selangor, Malaysia; E-Mail: rusliza@medic.upm.edu.my (R.B.); 3 Falkultas Farmasi, Universitas Sumatera Utara, 5 Jalan Almamater, USU-Kampus, Medan 20155, Indonesia; E-Mail: rosidah_noersal@yahoo.com (N.R.)

**Keywords:** anti-inflammatory activity, *Orthosiphon stamineus*, eupatorin, sinensetin, 3’-hydroxy-5,6,7,4’-tetramethoxyflavone, peritoneal capillary permeability

## Abstract

The aim of the present study was to verify the anti-inflammatory activity of *Orthosiphon stamineus* leaf extracts and to identify the active compound(s) contributing to its anti-inflammatory activity using a developed HPLC method. Active chloroform extract of *O. stamineus* was fractionated into three fractions using a dry flash column chromatography method. These three fractions were investigated for anti-peritoneal capillary permeability, *in vitro* nitric oxide scavenging activity, anti-inflammatory and nitric oxide (NO) inhibition using carrageenan-induced hind paw edema method. The flavonoid rich chloroform extract fraction (CF2) [containing sinensetin (2.86% w/w), eupatorin (5.05% w/w) and 3’-hydroxy-5,6,7,4’-tetramethoxyflavone (1.101% w/w)], significantly reduced rat hind paw edema, NO and decreased dye leakage to peritoneal cavity at p < 0.05. IC_50_ of *in vitro* NO scavenging of CF2 was 0.3 mg/mL. These results suggest that the anti-inflammatory properties of these CF2 may possibly be due to the presence of flavonoid compounds capable of affecting the NO pathway.

## 1. Introduction

*Orthosiphon stamineus* (OS), Benth. (Lamiaceae) has been used as a medicinal herb for many centuries in Southeast Asian countries including Indonesia and Malaysia. It is used as a remedy for catarrh of the bladder and as medicine for various disorders such as nephritis, nephrolithiasis, hydronephrosis, vesical calculi, arteriosclerosis, gout and rheumatism [1]. In Malaysia, the tea prepared from the leaves is taken as a beverage to improve general health and for the treatment of kidney disorders, bladder inflammation, gout and diabetes [2].

*Orthosiphon stamineus* contains several active constituents, such as terpenoids and polyphenols [3]. The therapeutic effects of OS have been ascribed mainly to its polyphenols, which are the most dominant constituents of the plant’s leaves [4]. Lipophilic flavonoids isolated from OS showed radical-scavenging activity towards diphenylpicrylhydrazyl radicals and inhibition of 15-lipoxygenase from soybeans used as a model for mammalian 15-lipoxygenase [5]. Some investigators have shown that the flavones sinensetin and 3’-hydroxy-5,6,7,4’-tetramethoxyflavone isolated from OS display diuretic activity in rats [6,7]. Diterpenes isolated from *Orthosiphonspp.* have been shown to exhibit anti-proliferative activities and suppressive effect on contractile responses in rat thoracic aorta [3,8,9,10], nitric oxide inhibition [11], cytotoxic effect against breast (MC-7) human cancer cell line, anti *Mycobacterium tuberculosis* [12] and anti-inflammatory activity induced by a tumor promoter 12-O-tetradecanoylphorbol-13-acetate (TPA) on mouse ears [13]. Recent studies have also shown that OS extract produces hepatoprotective effect against CCl_4_-induced hepatopathy [14] and hypoglycemic and anti-diabetes effects in normal and diabetic rats respectively [15]. One toxicological study has also shown that OS extract is less toxic and LD_50_ is >5,000 mg/kg [16]. Here we report the anti-inflammatory and nitric oxide inhibition of flavonoid rich chloroform fraction of the leaves of *O. stamineus* using different laboratory models.

## 2. Results and Discussion

Carrageenan-induced paw edema is an acute model of inflammation that has been reported to be effective in detecting orally-active anti-inflammatory drugs [17]. Development of edema induced by carrageenan is commonly associated with the early exudative stage of inflammation, one of the important processes of inflammatory pathology [18]. Carrageenan-induced edema is a multi-mediated phenomenon; include the liberation of a diversity of mediators. In the initial phase, there is bradykinin, histamine and serotonin liberated by local cells. After a couple of hours, there is a liberation of prostaglandins mediated by bradykinin, leukotrienes, and prostaglandins produced by macrophages [19,20]. 

**Figure 1:**
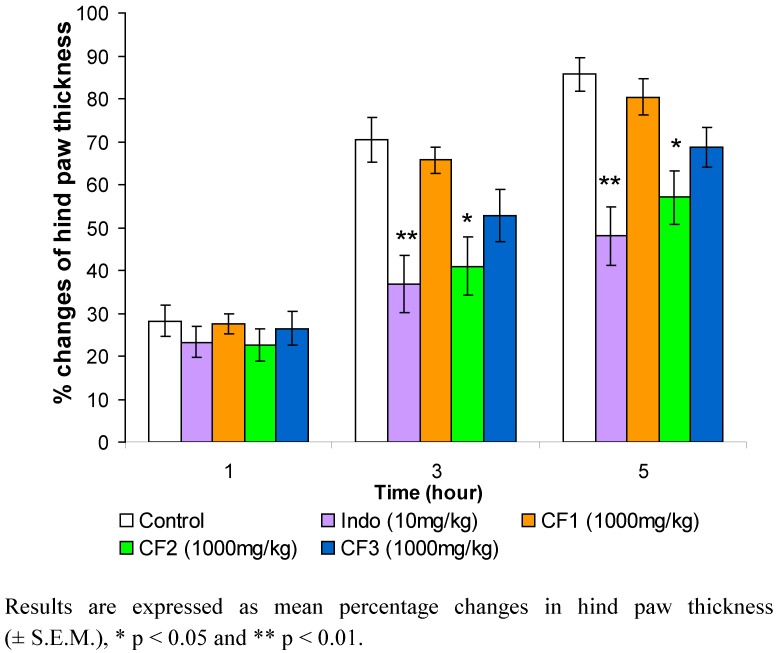
Effects of chloroform extract fractions of *Orthosiphon stamineus* leaf on carrageenan-induced rat hind paw edema (n=6).

In this experiment, the dried leaves of *O. stamineus* were successively extracted in a Soxhlet apparatus by the serial extraction method using petroleum ether followed by chloroform and finally with methanol. An anti-inflammatory screen of these three crude extracts showed that only the chloroform extract has a significant anti-inflammatory effect (data not shown). Hence, chloroform extract was further fractionated by chromatography into three fractions, namely, CF1, CF2 and CF3. The anti-inflammatory effects of these fractions were then studied. Only CF2 significantly reduced the hind paw edema in the third and fifth hours (Figure 1).

**Figure 2:**
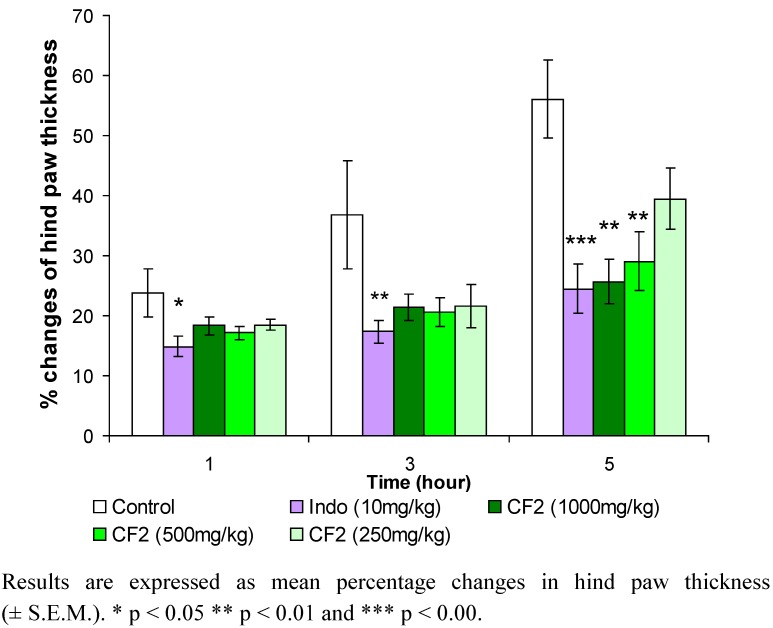
Effects of various doses of chloroform extract fraction 2 of *Orthosiphon stamineus* leaf on carrageenan-induced rat hind paw edema (n= 6).

**Figure 3:**
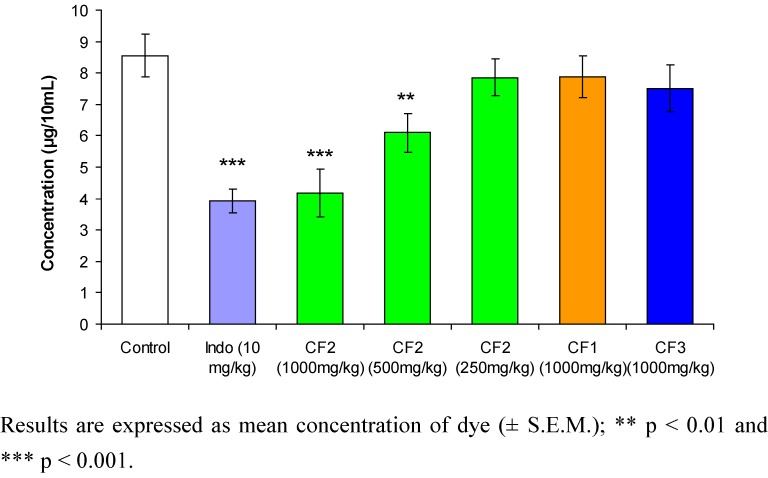
Effects of *Orthosiphon stamineus* leaf chloroform extract fractions on peritoneal capillary permeability (n=8).

The changes in hind paw thickness in the third hour for CF1, CF2, CF3 and indomethacin (used as standard) were 65.69%, 41%, 52.82% and 17.42%, respectively, while the changes in the fifth hour for CF1, CF2, CF3 and indomethacin were 80.35%, 57.06%, 68.70% and 17.8%, respectively. The dose-dependent pattern of CF2 is shown in Figure 2. The anti-inflammatory effect of CF2 decreases as the dose of the extract decreases. The results show that in the fifth hour, 1,000 mg/kg and 500 mg/kg doses of CF2 significantly reduced the hind paw edema at p < 0.01 compared with the control. The results are shown in Figure 3. CF2 significantly reduced the Chicago sky blue from leaking at the dose of 500 mg/kg (p < 0.01) and 1,000 mg/kg (p < 0.001) as compared to the control. Likewise, indomethacin (10 mg/kg) significantly reduced the dye leakage as compared to the control (p < 0.001).

The results obtained in the present study provide evidence that the CF2 was able to increase vascular permeability and possessed anti-inflammatory activity. At oral doses of 500 and 1,000 mg/kg, CF2 significantly prevented dye leakage into the peritoneal cavity and inhibited the edema induced in rats by carrageenan. The observed effect was quite similar to that exhibited by the group treated with indomethacin. The inflammatory response induced by carrageenan is, at least in part, due to prostaglandin release through arachidonic acid metabolism via the cyclooxygenase pathway. Several lines of evidence have shown a role for NO-related activation of COX in carrageenan-induced paw edema [20,21]. Carrageenan-induced edema has been found to increase the expression of iNOS and therefore contributes to the overproduction of NO, which is an effect has been shown to modulate hind paw inflammation process by increasing prostaglandin biosynthesis at the inflammatory site [23]. 

**Figure 4:**
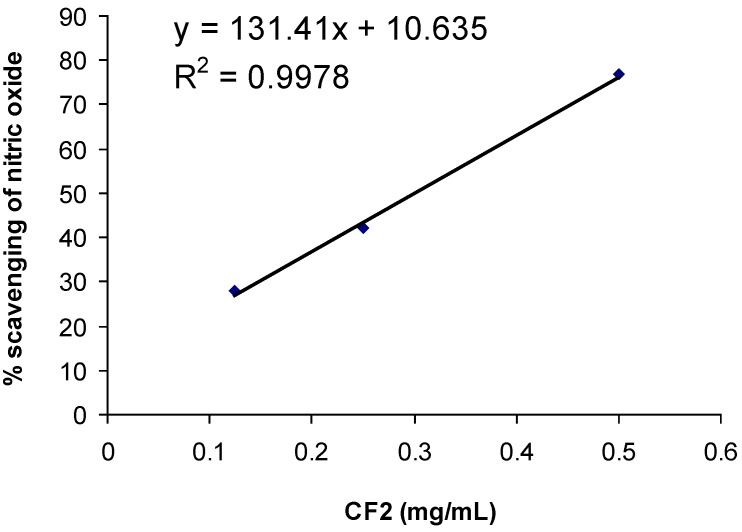
Effect of *Orthosiphon stamineus* CF2 on nitric oxide (NO) scavenging.

**Figure 5:**
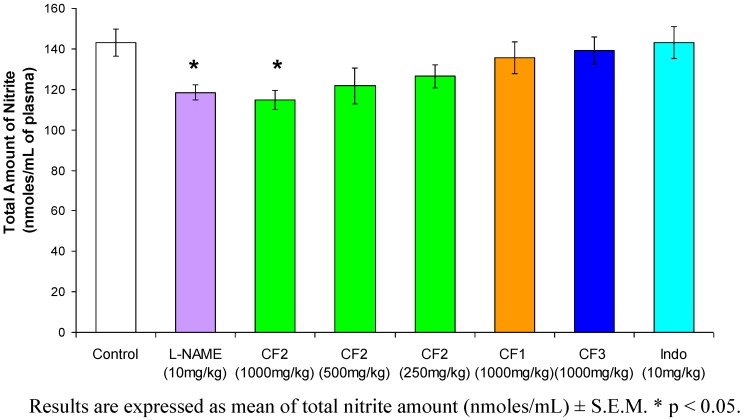
Effects of *Orthosiphon stamineus* leaf chloroform extract fractions on the total amount of nitrite in the plasma (n=6).

Our results clearly demonstrated that CF2 retained a NO-scavenging activity *in vitro* (IC_50 _= 0.3 mg/mL calculated according to the formula: y = 131.41x + 10.635) (Figure 4) and it significantly reduced NO plasma level *in vivo* (Figure 5) effects which were never seen with indomethacin. 

These findings support previous research indicating that OS was able to inhibit NO released from LPS-activated macrophage-like J774.1 cells [17]. While carrageenan mechanism of action does involve the activation of COX, it is logical to assume that CF2 could have modulated the inflammatory responses to carrageenan via firstly, the inhibition of NO and secondly, through the consequent inactivation of COX pathway and hence the inhibition of the synthesis and/or release of prostaglandins. 

**Figure 6:**
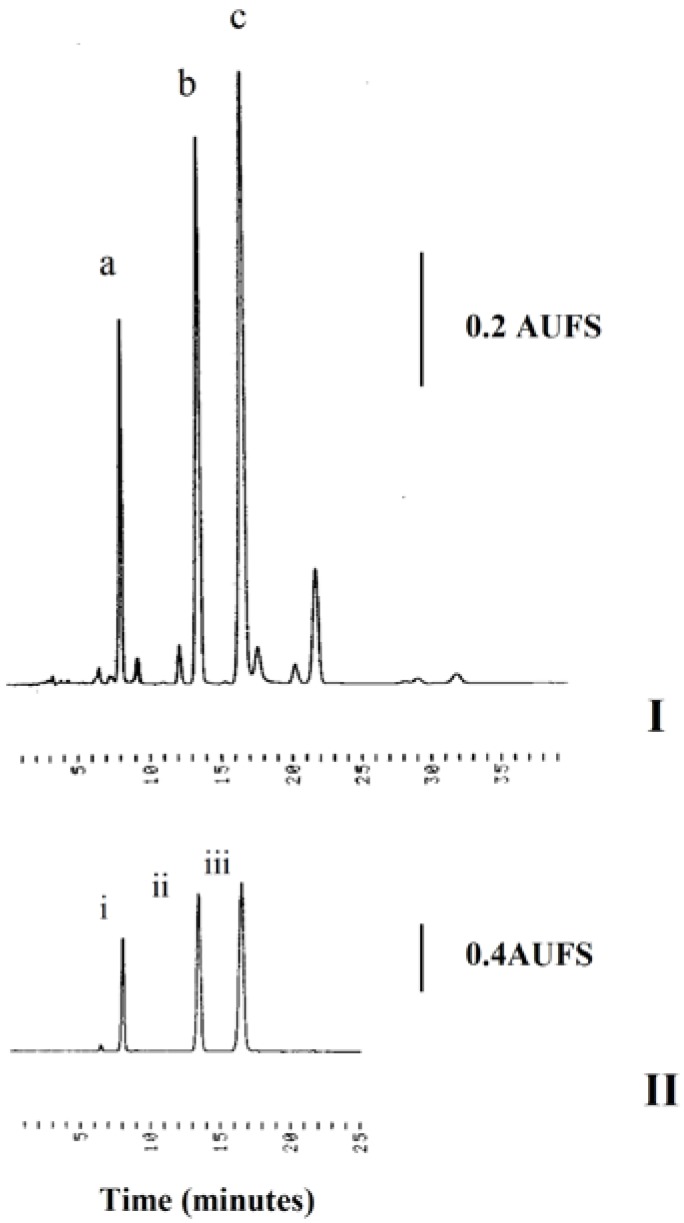
I) HPLC chromatogram of active sub-fraction CF2. Peaks: (a) TMF, (b) sinensetin, (c) eupatorin; II) HPLC chromatogram of standards. Peaks: (i) TMF, (ii) sinensetin, (iii) eupatorin.

Quantitative HPLC studies show that the concentrations of sinensetin, eupatorin and TMF determined from the calibration curves were 2.86, 5.05 and 1.01% (w/w), respectively, indicating eupatorin to be present in the highest amount in CF2, followed by sinensetin and lastly TMF (Figure 6). The regression equations of these curves and their coefficients of determination (R^2^) were calculated as follows: sinensetin: y = 5^7^x – 25599, R^2^ = 0.9998; eupatorin: y = 4^7^x + 66767, R^2^ = 0.9997; TMF: y = 4^7^x – 113138, R^2^ = 0.9975. The method shows a linear relationship between peak area and concentration over this range for these compounds. For the HPLC studies coefficient of variations for intra-day and inter-day studies were both less than 4.6% (Table 1 and Table 2). The precision as well as the reproducibility of this method were satisfactory. Considering as validation criteria for repeatability a relative standard deviations (RSD) not higher than 5%, on basis of results presented, the used method is precise. The recoveries of the spiked flavones namely eupatorin, sinensetin and TMF ranged from 96.23-102.02% with R.S.D. ranging from 0.83-4.43%. The newly developed HPLC method is thus validated for the quantification of flavonoid: TMF, eupatorin and sinensetin. In conclusion, this method is rapid, precise, reproducible, sample-saving and potentially useful for the quantitative analysis of other flavonoids.

## 3. Experimental Section

### 3.1. Materials

Carrageenan type IV (lambda), Chicago sky blue, indomethacin, L-NAME, nitrate reductase, β-nicotinamide adenine dinucleotide phosphate, phosphoric acid, sodium nitrate, sodium nitrite, sulfanilic acid and N-(1-naphtyl-1)-ethylenediamine dihydrochloride were purchased from Sigma (St. Louis, MO, USA). Carboxymethylcellulose (CMC), aluminum chloride, sodium chloride, sodium carbonate and sodium hydroxide were purchased from BDH Chemicals Ltd. (Poole, U.K.). Absolute alcohol, chloroform, petroleum ether, methanol and eosin were purchased from Riedel-de Haën (Seelze, Germany). Sinensetin, eupatorin and 3’-hydroxy-5,6,7,4’-tetramethoxyflavone were purchased from Indofine Chemical Co. (Hillsborough, NJ, USA). Silica gel-60 GF_254_, formaldehyde, TLC plate (silica gel-60 F_254_) and glacial acetic acid were purchased from Merck (Darmstadt, Germany). 

### 3.2. Experimental animals

Both female and male Charles River strain (ICR) mice and Sprague Dawley (SD) rats with body weights between 20-25 g and 180-250 g respectively, were used in this study. The animals were obtained from the Animal House, School of Pharmaceutical Sciences, Universiti Sains Malaysia. The animals were maintained at 28-30°C and allowed free access to food (normal laboratory chow, Gold Coin) and tap water *ad libitum*. All the experimental animals were fasted 14-16 hours prior to the experiments. The animals were acclimatized to laboratory condition for seven days before commencement of experiments. The experiments were approved by the Animal Ethics Committee, Universiti Sains Malaysia.

### 3.3. Plant material

Plants were grown from cuttings, using standard agronomic practices, at Kepala Batas, Penang, Malaysia. The leaves were collected in the late afternoon from white-flowered plants. A voucher specimen (10106) was deposited at the herbarium of School of Biology, and a voucher specimen (027) was deposited at Bilik Herba, School of Pharmaceutical Sciences, Universiti Sains Malaysia. 

### 3.4. Successive soxhlet-extraction and fractionation of chloroform extract

The plants were dried in an oven (Memmert, Germany) at 40°C. Leaves were separated and then ground into powder using a Bench Top Crusher RT-34 with 120 mesh perforated sieves (Rong Tsong Precision Technology Co., Taiwan) and subsequently extracted in a Soxhlet apparatus by the serial extraction method, using petroleum ether, followed by chloroform, and finally with methanol (yields of 4%, 4.9% and 12.2%, respectively). The extracts were concentrated using a Büchi-RE121 evaporator (Büchi Laboratorium-Technik AG, Switzerland) equipped with a Büchi-B169 vacuum system, and then dried in a Hetovac VR-1 (HETO Lab Equipment, Denmark) freeze dryer. The lyophilized extract was then kept in desiccators at room temperature (22-24 °C) prior to use in our experiments. 

A suspension of silica gel-60 GF_254_ (Art No. 7730, 70g) was packed into a sintered glass column measuring 27 cm long and 5 cm in diameter and fitted with a stopcock. The column was tapped gently to allow the powder to settle and to remove any voids. Suction was first applied gently to the column and then in full with a water aspirator. Subsequently, the silica was pressed carefully with the flat bottom of a flask, first around the circumference and then towards the center to produce a totally level, well-compacted bed and headspace for the addition of the mixture and eluent. The column was then pre-eluted under vacuum with 250 mL of petroleum ether to give a well-packed column [25]. 

Based on anti-inflammatory screening results (data not shown), the crude chloroform extract was found to be the most active and therefore subjected for further fractionation. Crude chloroform extract (10 g) was pre-adsorbed onto the adsorbent by first dissolving the extract in 200 mL of chloroform. Silica gel 7730 (20 g) was then added to a solution containing the chloroform crude extract and the solvent evaporated off from the sample using a rotary evaporator. The resultant dried sample mixture was then placed onto the top of the dry-column for flash chromatography and packed evenly by applying suction. The column was first eluted with 2 × 250 mL of 100% petroleum ether followed successively by 2 250 mL of petroleum ether-chloroform (1:1); 2 × 250 mL petroleum ether-chloroform (3:7); 2 × 250 mL of 100% chloroform; 2 × 250 mL of chloroform-methanol (8:2); 2 × 250 mL of chloroform-methanol (1:1); 2 × 250 mL chloroform-methanol (3:7); 2 × 250 mL of chloroform-methanol (1:9) and finally 3 × 250 mL of 100% methanol. The fractions were examined by thin layer chromatography and those giving the same profiles were pooled together, affording three fractions labeled as CF1, CF2, and CF3 yield 18%, 16.3% and 41.4% of CE respectively. 

### 3.5. Carrageenan-induced hind paw edema test

A modification of the method described by Winter *et al*. was used [26]. Male and female Sprague Dawley rats were injected in the sub-plantar region of the right hind paw with 1% carrageenan solution in 0.9% sodium chloride (NaCl) (w/v) (0.1 mL). The test drug and fractions were suspended in 1% carboxymethylcellulose (CMC) in distilled water and 10 mL/kg was administered orally by gavage an hour before carrageenan injection. The animals in the control group received 1% of CMC in distilled water. The footpad thickness was measured by placing the unanesthetized animal foot between the anvil and spindle of peacock dial thickness gauge (micrometer) (Ozaki Ltd., Japan) before, and 1, 3 and 5 hours after injection of carrageenan. The percentage of changes in hind paw thickness was calculated according to the following formula:

% increase = (C_t _–C_0_)/C_0_ × 100

where C_t_ = thickness of hind paw at *t* hour after carrageenan injection; and C_0_ = thickness of hind paw before carrageenan was injection.

### 3.6. Acetic acid-induced peritoneal capillary dye leakage test in mice

The capillary permeability or dye leakage test was carried out by the modified method of Whittle [27]. Male and female ICR mice were given the treatments of chloroform extract’s fractions and drug. Control group mice were given 1% CMC in distilled water orally. After 25 minutes, each animal was given an intravenous injection of 4% solution of Chicago sky blue (in normal saline, 0.1 mL). Thirty minutes later, 0.25% (v/v) glacial acetic acid in normal saline (0.2 mL) was injected peritoneally. Twenty minutes after injection the mice were killed by dislocation of the neck, and the viscera organs were exposed. After allowing blood to drain away from the abdominal wall and jugular vein for 1 min, the animals were held by a flap of the abdominal wall, and the viscera organs were irrigated with distilled water in a Petri dish. The combine washings were filtered through glass wool and filled up a 10 mL in a volumetric flask. Then 0.1 N sodium hydroxide solution (0.1 mL) was added to each tube in order to clear any turbidity due to protein. The absorbance was read at 590 nm with a Hitachi U-2000 spectrophotometer (Hitachi, Japan). The total concentration of the Chicago sky blue was determined as ng/10 mL from a standard calibration curve.

### 3.7. Carrageenan-induced nitric oxide

Carrageenan suspension (0.1 mL, 1%) was injected in the right hind paw of each rat under the sub-plantar region. Rats were orally administered with the chloroform extract’s fraction 1 h before carrageenan injection. Control rats received an equal volume (10 mL/kg) of saline, while L-NAME (10 mg/kg), a nitric oxide (NO) inhibitor, was used as reference drug. The rats were anesthetized at the 4^th^ hour after injection of carrageenan. Blood was collected by cardiac puncture before the rats were killed. Plasma was prepared by centrifuging blood at 3,000 rpm for 10 min at 4 ºC and stored at -70 ºC to measure total nitrite by the Griess assay [28].

### 3.8. Griess assay

The Griess reaction used for the assay of nitrite was adapted from previously described method [28]. Standard curves for both sodium nitrite and sodium nitrate were prepared. Sixty μL samples were treated with nitrate reductase (5 unit/mL, 10 μL) and 30 μL NADPH (β-nicotinamide adenine dinucleotide phosphate) (1.25 mg/mL). Two hundred μL of Griess reagent (5% phosphoric acid, 1% sulfanilic acid and 0.1% *N*-(1-naphtyl-1)-ethylenediamine dihydrochloride) was then added and proteins were subsequently precipitated by 10% trichloroacetic acid (200 μL). Tube contents were vortex mixed then centrifuged at 13,000 rpm. Duplicate 200 μL samples of supernatants were transferred to a 96-well flat bottomed microplate and absorbance read at 540 nm using a Power Wave X340 microplate reader (Bio-Tek instrument Ing., USA). Values for the concentration of nitrite assayed were calculated from standard calibration plots for sodium nitrate and sodium nitrite. 

### 3.9. Assay for nitric oxide scavenging activity

A modified method from Ravishankara *et al.* was adapted in this experiment [29]. Sodium nitroprusside (10 mM) in phosphate buffer solution (PBS) (pH 7.4) (1 mL) was incubated with various concentrations (0.5, 0.25, 0.125 and 0.0625 mg/ml) of CF2 dissolved in methanol (1 mL), and incubated at room temperature (24-26 ºC) for 150 min. After the incubation period, Griess reagent (1 mL, 2% phosphoric acid, 1% sulphanilic acid, 0.1% *N-*(1-napthyl-1)-ethylenediamine in 100 mL deionized water) was added. The absorbance of the chromophore formed was read at 546 nm. Percentage inhibition (Q) was calculated with the formula:

Q = 100(A0 – Ac)A0

where A0 = the absorbance of sodium nitroprusside in PBS after 150 min incubation; Ac = the absorbance of CF2 with nitroprusside in PBS after 150 min incubation; The IC_50_ (inhibition concentration of 50%) value was calculated with the equation of ICx = (x-10.635)/131.4, where x = percentage of inhibition.

### 3.10. HPLC analysis

HPLC analysis was performed on a system consisting of a Gilson model 302 pump, Rheodyne 7125 injector, a Linear model UVIS 204 detector set at 340 nm, guard column, a reversed-phase C18 column (Purospher^®^STAR, 5 µm, 250 mm × 4.6 mm i.d.) and a Hitachi D-2500 Chromato Integrator. The mobile phase used was acetonitrile-water = 40:60 set at a flow rate of 1.2 mL/min. Standard solutions and sample were prepared by weighing an accurate amount of 3’-hydroxy-5,6,7,4’tetramethoxyflavone (TMF), eupatorin and sinensetin (0.1, 0.05, 0.025, 0.0125, 0.00625 mg respectively) and CF2 (1 mg) and dissolving each of them in methanol (1 mL). The standard solutions were injected into the HPLC for analysis. Calibration graphs of the peak areas against concentrations were subsequently plotted for linear regression analysis.

#### 3.10.1. Recovery studies

A fixed amount of eupatorin or sinensetin or TMF was added separately to the sample CF2 solution and mixed to ensure dissolution. The resultant solution was then injected into HPLC. The recovery of each component was determined using the following formula [30]:

Recovery (%) = [ ( A-B ) / C ] × 100%

where A is the detected amount of eupatorin or sinensetin or TMF in the spiked sample, B is the detected amount of eupatorin or sinensetin or TMF in CF2 without spiking, and C is the added amount of the eupatorin or sinensetin or TMF.

**Table 1:** Recovery of sinensetin, eupatorin and TMF.

Standard	Amount added (mg)	Recovery	
		Mean (%)	R.S.D. (%)
Sinensetin	0.17	101.5	0.83
	0.13	97.73	4.07
	0.04	98.10	1.57
Eupatorin	0.20	97.41	3.76
	0.10	96.23	4.43
	0.05	102.0	2.30
TMF	0.19	97.54	2.76
	0.11	98.43	1.99
	0.06	99.23	3.89

#### 3.10.2. Precision studies

The precision (repeatability) study of the method was carried out using standard solutions of these flavones injected five times each on the same day and over a 5-day period [28].

**Table 2:** Intra-day and inter-day precision for determination of sinensetin, eupatorin and TMF.

Standard	Concentration (µg/mL)	R.S.D. (%)	
		Intraday (n = 5)	Interday (n = 5)
Sinensetin	100	2.54	3.67
	50.0	4.35	3.10
	25.0	2.13	2.67
	12.5	1.27	2.17
	6.25	4.52	4.09
Eupatorin	100	4.05	4.25
	50.0	1.30	4.52
	25.0	1.56	4.15
	12.5	3.70	2.42
	6.25	1.69	3.10
TMF	100	2.43	3.12
	33.3	1.45	3.54
	11.1	3.21	3.98
	3.70	1.98	2.99
	1.23	3.67	3.45

### 3.11. Isolation and structure determination of eupatorin and sinensetin

The CF2 fraction (0.2 g) was subjected to silica gel chromatography and eluted with chloroform/ethanol (85:15). The smaller fractions were combined and further purified by preparative TLC on silica gel 60 F_254_ precoated plate (10 × 20 cm) to give eupatorin (8.9 mg) and sinensetin (4.2 mg) for spectroscopic analysis determination. Rotary evaporator including vacuum pump and water bath from Buchi (Switzerland) were used for evaporating off the solvent. IR spectra recorded in KBr were taken on Nexus FT-IR spectrophotometer; UV spectra for the compounds dissolved in methanol were determined using a Hitachi U-2000 UV-visible spectrophotometer; ^1^H-NMR and ^13^C-NMR spectra were recorded in CDCl_3_ on Bruker AC 400 (400 MHz for ^1^H-NMR and 100 MHz for ^13^C-NMR). The GC was performed using an Agilent 6890 series with split injector and fused silica capillary column [ a non polar column, Scientific Glass Engineering (SGE) Australia], 60m × 0.25 mm, i.d., with a HP 5 methysilicone film (film thickness 0.25 µm). The conditions were as follows; injector temperature, 250 ºC; carrier gas, Helium; column flow rate, 0.9 mL/min; splitting ratio, 10:1; injection volume, 2 µL in all experiment; oven temperature was programmed from 70 ºC to 320 ºC at 22.5 ºC/min. The total ion chromatograms (TICs) and mass spectra were recorded using an Agilent Network 5973N mass engine with enhanced chemstation in the electron impact ionization mode at 2200 eV. The transfer line is maintained at 280 ºC, the source temperature at 230 ºC (maximum 250 ºC) and quadrupole temperature at 150 ºC (maximum 200 ºC). All melting points (uncorrected) were measured with a Gallenkamp (England) melting point apparatus.

*Eupatorin*: Yellow powder. Mp: 197-199°C. GC-MS : 344 [M^+^]. FTIR (KBr): 3430 cm^-1^ (OH), 2945 cm^-1^ (CH), 1653 cm^-1^ (C=O), 1497-1566 cm^-1^ (C=C aromatic) 1605 (C=C stretch). ^1^H-NMR (CDCl_3_) δ: 6.58 (1H, s, 3-H), 12.78 (1H, s, 5-OH), 6.61 (1H, s, 8-H), 7.45 (1H, m, 2’-H), 5.83 (1H, s, 3’-OH), 6.98 (1H, d, *J* = 8.49 Hz, 5’-H), 7.48 (1H, s, 6’-H), 3.96 (3H, s, 6-OMe), 4.00 (3H, s, 4’-OMe), 4.02 (3H, s, 7-OMe). ^13^C-NMR (CDCl_3_) δ: 164.2 (C2), 104.9 (C3), 182.6 (C4), 153.4 (C5), 133.8 (C6), 158.5 (C7), 90.0 (C8), 153.1 (C9), 104.7 (C10), 124.9 (C1’), 111.1 (C2’), 146.4 (C3’), 149.9 (C4’), 112.7 (C5’), 119.4 (C6’), 61.2 (OMe)*, 56.6 (OMe)*, 56.6 (OMe)*. 

**Figure 7:**
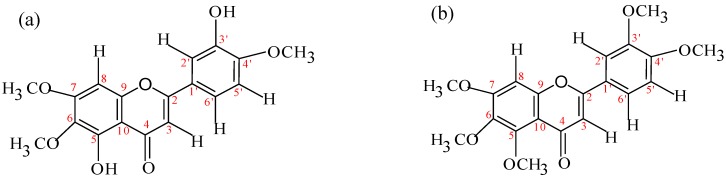
Chemical structure of isolated compounds. (a) 5,3’-dihydroxy-6,7,4’-trimethoxy-flavone (eupatorin), (b) 5,6,7,3’,4’-pentamethoxyflavone (sinensetin).

*Sinensetin*: Colorless solid. Mp: 174-176°C. GC-MS: 372 [M^+^]. FTIR (KBr): 2996 cm^-1^ (CH), 1633 cm^-1^ (C=O), 1450=1546 cm^-1^ (C=C aromatic). ^1^H-NMR (CDCl_3_) δ: 6.61 (1H, s, 3-H), 6.81 (1H, s, 8-H), 7.35 (1H, s, 2’-H), 6.99 (1H, d, *J* = 8.54 Hz, 5’-H), 7.52 (1H, d, *J* = 8.47 Hz, 6’-H), 4.00 (3H, s, 3’-OMe), 3.98 (3H, s, 4’-OMe), 3.89 (3H, s, 5-OMe), 3.94 (3H, s, 6-OMe), 4.01 (3H, s, 7-OMe). ^13^C-NMR (CDCl_3_) δ: 162.1 (C2), 108.4 (C3), 178.1 (C4), 152.9 (C5), 141.4 (C6), 157.8 (C7), 97.3 (C8), 154.8 (C9), 108.1 (C10), 121.2 (C1’), 109.8 (C2’), 149.9 (C3’), 150.3 (C4’), 112.2 (C5’), 120.6 (C6’), 63.2 (OMe)*, 62.5 (OMe)*, 57.3 (OMe)*, 57.2 (OMe)*, 57.1 (OMe)* (* these assignments may be interchanged). Eupatorin and sinensetin gave a positive TLC flavone test which showed a dark blue colouration after being sprayed with NP/PEG and observed under UV 365 nm (data not shown). The UV spectrum showed λ_max_ (log ε) at 243 nm (1.06) and 343 nm (1.36) for eupatorin [Figure 7(a)] and 307 nm (1.05) and 334 nm (1.32) for sinensetin [Figure 7(b)], confirmed the presence of a flavone skeleton. 

### 3.12. Experimental design and analysis of data

Randomized Complete Block Design was used in the experiments. Data were expressed as means (± S.E.M.). Two-way analysis of variance (ANOVA) followed by Dunnett Multiple Comparison Test was used to compare treatment groups and control group by SPSS (statistical package for social sciences) version 10.0. 

## 4. Conclusions

The results shown suggest that the anti-inflammatory activity of CF2 is largely attributable to the presence of some bioactive compounds such as the flavonoids sinensetin, eupatorin and TMF [contents: 2.86, 5.05 and 1.101% (w/w), respectively]. Such anti-inflammatory properties may be ascribed to inhibition of prostaglandin synthesis and reduced NO production [24]. It is important to point out that work is in progress to investigate the anti-inflammatory activity of the isolated compounds present in CF2 fraction of *Orthosiphon* stamineus leaf.
